# The role of insulin-like growth factor in Acrochordon Etiopathology

**DOI:** 10.1186/s12895-020-00111-0

**Published:** 2020-11-03

**Authors:** Hanife Gündoğdu Köseoğlu, Burçin Cansu Bozca, Cumhur İbrahim Başsorgun, Ramazan Sarı, Sadıka Halide Akbaş, Ayşe Akman Karakaş

**Affiliations:** 1 Akdeniz Şifa Hospital, Antalya, Turkey; 2 Dermatology and Venereology Department, Akdeniz University School of Medicine, Antalya, Turkey; 3 Pathology Department, Akdeniz University School of Medicine, Antalya, Turkey; 4 Endocrinology and Metabolism Department, Akdeniz University School of Medicine, Antalya, Turkey; 5 Biochemistry Department, Akdeniz University School of Medicine, Antalya, Turkey

## Abstract

**Background:**

There are reports that acrochordon (skin tag), the most common fibroepithelial tumor of the skin, may be associated with metabolic syndrome components, particularly insulin metabolism disorders. However, to the best of our knowledge, there is no study examining its association with insulin resistance and tissue levels of insulin-like growth factor 1 receptor (IGF-1R) and insulin-like growth factor 2 receptor (IGF-2R).

**Methods:**

Thirty patients with at least one acrochordon in their body who had no known history of diabetes mellitus and a control group comprised 30 individuals who had no acrochordon or no known history of diabetes mellitus were included. The tissue expression of IGF-1R and IGF-2R were investigated via immunohistochemical assessment in both groups.

**Results:**

In the group with acrochordon, IGF-1R and IGF-2R expression was found to be significantly higher compared to the control group (*p* < 0,01). Using logistic regression analysis, an increase in serum insulin, serum IGF-1 and HOMA-IR levels was found to be associated with the expression levels of IGF-1R and IGF-2R.

**Conclusion:**

These findings support the view that insulin metabolism disorders should be evaluated in patients with acrochordon. Our study indicates that IGF receptors may have an effect on acrochordon pathogenesis and that acrochordon etiology and related conditions can be clarified by detection of parameters that influence receptor levels.

## Background

Acrochordons are 1 mm to 1 cm (rarely giant sized) papules that may or may not have stems. Their color ranges from skin color to dark brown [1]. Although acrochordon development has been associated type 2 diabetes mellitus (DM), glucose intolerance, hyperinsulinemia, obesity, pregnancy, acromegaly, aging, colonic polyps and genetic predisposition, its cause has not been fully understood [2, 3].

Insulin-like growth factor (IGF) 1 and 2 are anabolic proteins that are involved in growth and they also mediate the emergence of many of the anabolic and mitogenic effects of growth hormone [4]. Three different IGF receptors have been identified; insulin receptor, IGF–1R, and IGF–2R [5]. The insulin receptor and IGF–1R are approximately 60% similar with regard to amino acid structure. IGF–1R has an essential role in cell growth and differentiation [6]. Hyperinsulinemia increases the serum level of free IGF–1 and decreases the serum level of insulin-like growth factor binding protein (IGFBP)–3. Hyperinsulinemia and increased IGF-1 levels directly result in the growth of epithelial and fibroblastic cells via receptor activation. This association explains the relatively frequent development of acrochordon and pseudoacanthosis nigricans in hyperinsulinemic patients [7].

The signal cascade following the activation of the IGF-2R receptor is not fully known. It is believed that the task of the IGF–2R receptor is to decrease the serum level of IGF-2 which is the only ligand of this receptor. When the level of IGF–2 decreases in the serum, it binds to IGF-1R to a lesser extent. Thus, IGF-2R is thought to suppress tumor growth and proliferation by indirectly suppressing IGF-1R activation [8].

In contrast to the results of studies investigating the effects of DM and dyslipidemia on acrochordon development, there are no studies examining insulin resistance, IGF-1R and IGF-2R levels at the tissue level in this context. In this study, we aimed to investigate IGF–1R and IGF–2R levels in acrochordon tissue, as well as metabolic disease indicators.

## Methods

A total of 30 patients aged between 21 and 66 years admitted to the Dermatology outpatient clinic of Akdeniz University School of Medicine Hospital during a 6-month period, who had least one acrochordon lesion and no known diabetes mellitus, were enrolled in the study. The control group consisted of 30 healthy age- and gender-matched individuals, aged between 18 and 77 years, who had no acrochordons and were free of diabetes mellitus. The study was approved by the local ethics committee of our hospital. All participants signed an informed consent form.

The location, number, dimensions, shape and edge properties of the acrochordons, as well as the surface and base shape, sensorial features (itching, discomfort) and duration of the lesions, were recorded. Body mass index (BMI = Body weight/height2 [kg/m2]) of patients with acrochordon and control group was calculated. The BMI results were evaluated according to the World Health Organization (WHO) classification for excess weight. Venous blood samples were taken following 12 h of fasting, and biochemical analysis of fasting blood glucose, total cholesterol, triglyceride, LDL cholesterol, HDL cholesterol, basal insulin, IGF-1, HbA1c, leptin, and free fatty acid levels were measured in the serum. Additionally, all participants underwent an oral glucose tolerance test (OGTT) and their homeostatic model assessment-insulin resistance (HOMA-IR) values were calculated.

Venous blood samples were taken following 12 h of fasting. Fasting blood glucose, total cholesterol, triglyceride, LDL and HDL cholesterol levels were measured by enzymatic colorimetric methods with a Modular PPP autoanalyser (Roche Diagnostics, GmbH, Mannheim). Whole blood HbA1c levels were measured by turbidimetric immunoinhibition assay (TINIA) with the same device (Roche Diagnostics, GmbH, Mannheim). Fasting insulin levels were determined by electrochemiluminescence immunoassay (ECLIA) method in an E–170 immunoanalyser (Roche Diagnostics, GmbH, Mannheim). Serum IGF-1 and leptin levels were measured by commercially available ELISA kits. Serum non-esterified (free) fatty acid (FFA) levels were determined with an automated enzymatic colorimetric method (NEFA-HR, Wako Diagnostics, USA). A 2-h, 75-g oral glucose tolerance test (OGTT) was administered to all patients.

Tissue specimens were taken from the 30 patients with acrochordon lesions by superficial excision after local anesthesia. The control group for pathological examination was formed by selecting 30 non- tumoral skin tissue samples from the intact tissue archive of the Pathology Department. Tissue samples were fixed with formalin and embedded in paraffin. Sections of 4–5 μm thickness were prepared from these paraffin blocks and immunohistochemically exposed to IGF–1R (sc1422 IGF–1[G–17], Santa Cruz Biotechnology) and IGF–2R (GTX28093 IGF2 Receptor antibody, Genetex) antibodies (1:100 and 1:50, respectively) via the ‘Streptavidin-Biotin complex’ method. Briefly, tissue sections were incubated at 56 °C overnight, and then paraffin was removed with xylol (two applications with 5 min of duration). Then, they were passed through decreasing concentrations of alcohol and rehydrated with distilled water. The antigen retrieval process was applied for 30 min in a hot (90 °C) water tank containing EDTA buffer at pH = 8.0, followed by cooling for 20 min and a wash in phosphate buffered saline. The sections were then incubated for 10 min with 3% hydrogen peroxide solution to perform endogenous peroxidase enzyme blocking. After being allowed to stand in PBS for 20 min to prevent background staining, the sections were incubated overnight at room temperature with the aforementioned primary antibodies for IGF-1R and IGF-2R. Following the incubation, treatment with ‘linking reagent’ was performed for 15 min and a final incubation with streptavidin conjugated ‘horseradish peroxidase’ was performed for 15 min. The staining was made visible with diaminobenzidine and covered with lamella after contrast staining with hematoxylin. Histological evaluation was done semi-quantitatively by light microscopy. Cytoplasmic and membranous staining was considered positive and these results were recorded on a present/absent basis. We also determined staining intensity with the following grading: negative (−), mild (+), moderate (++) and intense (+++). All immunohistochemistry analyses were performed routinely by the personnel of the Pathology Department who were blinded to the study plan and patient diagnoses.

### Statistical analysis

The data was analyzed with the SPSS version 18.0 software for Windows. The differences between study groups in terms of categorical variables were tested by the Fisher’s exact test or Pearson Chi-Square test. Numerical variables were compared with the Mann-Whitney-U or Student t-test depending on normality of distribution (tested with Shapiro Wilk). The correlations between two independent variables was tested with the calculation of Spearman correlation coefficient, and factors affecting the development of acrochordon were analyzed and interpreted by logistic regression analysis methods. A *P* value of < 0.05 was considered statistically significant.

## Results

Of the 30 patients included in the study group, 18 (60%) were female and 12 (40%) were male. The age of patients with acrochordon ranged between 21 and 66 years, with a mean age of 48.17 ± 12.08 years. The control group consisted of 17 women (56.7%) and 13 men (43.3%). The age of patients in the control group ranged between 18 and 77 years, with a mean age of 48.37 ± 13.81 years. There was no statistically significant difference between the two groups in terms of age and sex distribution (*p* = 0.953 and *p* = 0.793, respectively). The disease duration of the group with acrochordon ranged from 1 to 35 years, with a mean duration of 7.9 ± 7.07 years (median = 5 years). Twenty-three of the patients with acrochordon had lesions in multiple locations.

The mean BMI value was 30.55 ± 5.09 kg/m2 in the acrochordon group, and 28.17 ± 6.19 kg/m2 in the control group. The mean BMI of the study group with acrochordon was significantly higher than that of the control group (*p* = 0.040). In the patient group, there was no significant correlation between current acrochordon count and BMI value (*p* = 0.206).

When we examined the fasting blood glucose (FBG) level of the study group 9 (30%) patients had impaired FBG levels in the patient group and 17 (56.7%) subjects had impaired FBG levels in the control group (*p* = 0.060). There were also no statistically significant difference between the groups with regard to OGTT results (*p* = 0.16).

The mean HbA1c value was 5.83 ± 0.62% in the acrochordon group and 6.06 ± 0.67% in the control group (*p* = 0.037). Mean serum insulin level was 13.87 ± 12.53 uU / ml in the acrochordon group and 10.45 ± 9.61 uU / ml in the control group (*p* = 0.031). The mean HOMA-IR value of the acrochordon group was slightly higher but statistically similar to controls (*p* = 0.069).

There were no statistically significant differences between the control group and acrochordon group in terms of triglyceride, total cholesterol, VLDL, HDL, LDL cholesterol, FFA levels and total cholesterol/HDL and LDL/HDL ratios (*p* > 0.05, for all). The mean serum leptin level was 11,228.97 ± 4.56 pg / ml in the acrochordon group and 9970.30 ± 4.49 pg / ml in the control group (*p* = 0.352).

The mean serum IGF-1 value was 63.89 ± 40.96 ng / ml in the acrochordon group and 92.3 ± 73.98 ng / ml in the control group (*p* = 0.193) In addition, there was no significant correlation between serum IGF-1 level and acrochordon count (*p* = 0.671).

**Fig. 1:**
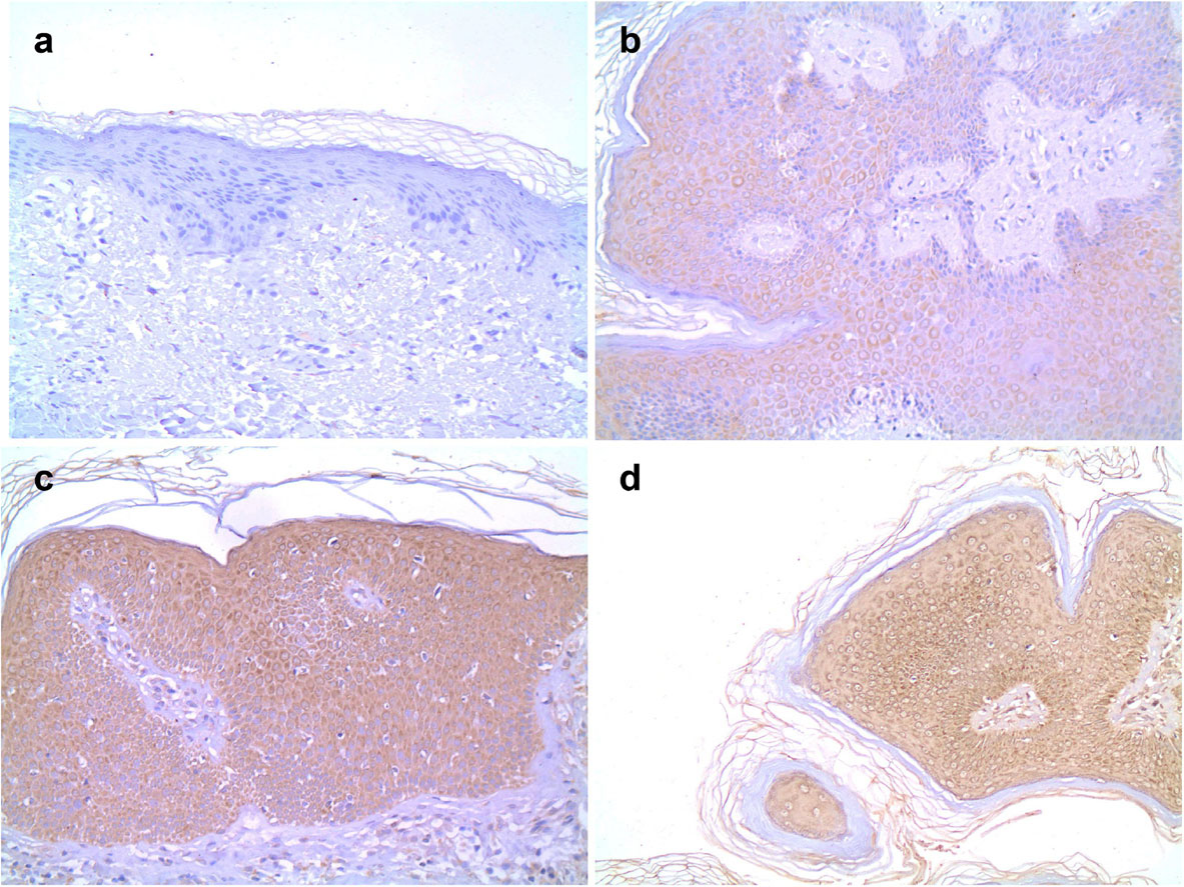
IGF-1R staining. **a**. negative IGF-1R staining in normal tissue (1 × 100) **b**. mild (+) staining with IGF-1R in acrochordon tissue (1 × 100) **c**. moderate (++) staining with IGF-1R acrochordon tissue (1 × 200) **d**. intense (+++) staining with IGF-1R in acrochordon tissue (1X100) Scale bar: 50 μm

**Fig. 2:**
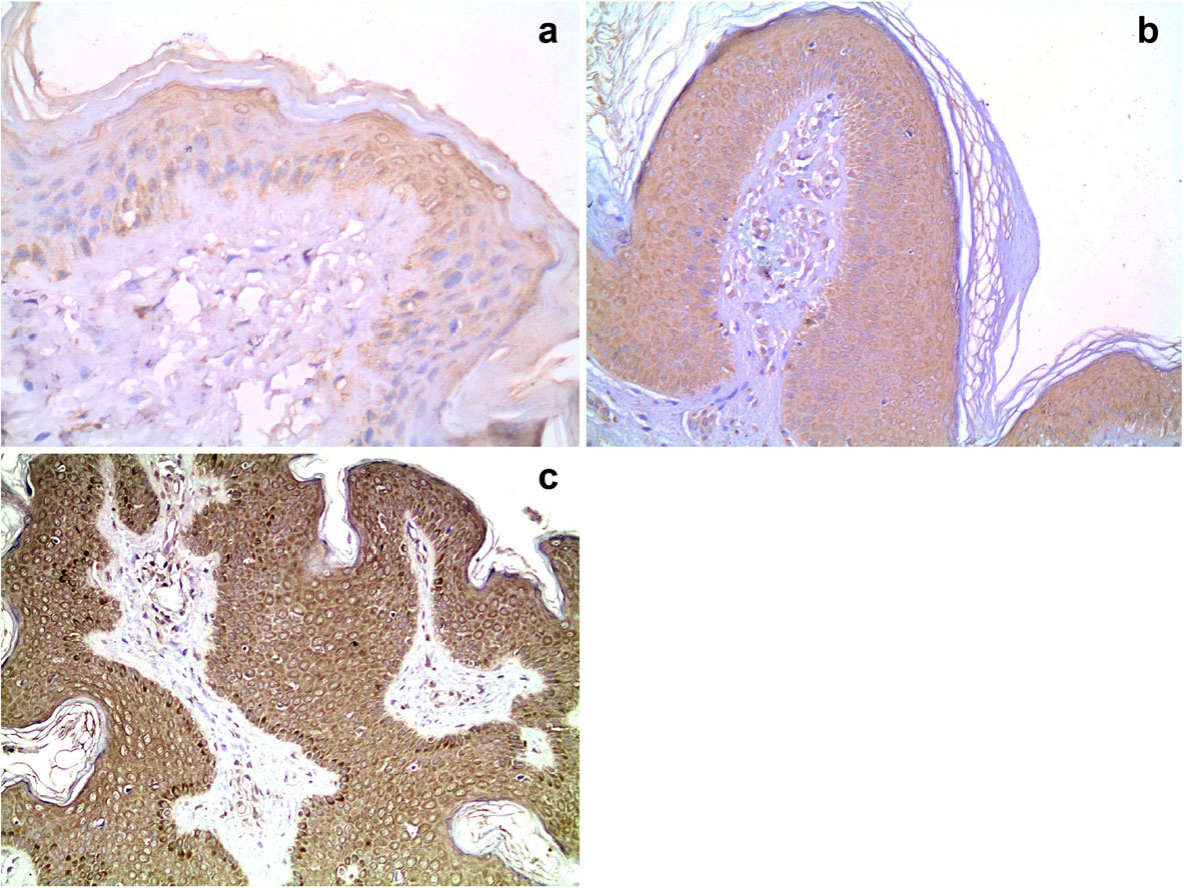
IGF-2R staining in acrochordon tissue. **a**. Mild (+) staining with IGF-2R in acrochordon tissue (1 × 200) **b**. Moderate (++) staining with IGF-2R in acrochordon tissue (1 × 100) **c**. Intense (+++) staining with IGF-2R in acrochordon tissue (1 × 100) Scale bar: 50 μm

Positive staining for IGF-1R and IGF-2R was identified in almost all patients with acrochordon (*p* < 0.001 for both). Only 5 controls had positive staining for IGF-1R (mild staining) and only 6 had staining for IGF-2R (Table 1). Negative IGF-1R staining in normal tissue is shown in Fig. 1a, IGF-1R with mild (+), moderate (++) and severe (+++) staining in Fig. 1b, c and d, respectively. An image showing mild (+), moderate (++) and intense (++) staining with IGF-2R in acrochordon tissue is shown in Fig. 2.

**Table 1:** The distribution of IGF-1R and IGF-2R staining levels according to groups

	No staining (−) n (%)	Mild (+) n (%)	Moderate (++) n (%)	Intense (+++) n (%)
IGF-1R staining				
**Patients**	1 (3.3)	12 (40)	14 (46.7)	3 (10)
**Controls**	25 (83.3)	5 (16.7)	0 (0)	0 (0)
IGF-2R staining				
**Patients**	–	1 (3,3)	5 (16,7)	24 (80)
**Controls**	24 (80)	6 (20)	–	–

The levels of IGF-1R and IGF-2R staining did not demonstrate any relationships with gender, age, the number and localization of lesions, lesion duration and HOMA-IR. However, the frequencies of positive staining with IGF-1R and IGF-2R were significantly higher in normoglycemic patients with acrochordon compared to controls (p < 0.01). Although the relationships were minor, we found that the intensity of IGF-1 staining was correlated with serum IGF-1 levels (r = 0.028, *p* = 0.003) and BMI (r = 0.257, *p* = 0.048). On the other hand, when staining intensity was assessed, we found that IGF-2R staining intensity was significantly correlated with BMI (r = 0.375, p = 0.003) and serum insulin levels (r = 0.27, *p* = 0.037).

In logistic regression analysis, independent factors associated with the presence of acrochordon were investigated. None of the variables included in the model (BMI, serum insulin levels, IGF-1R and IGF-2R staining) were found to be independently associated with the presence of acrochordon (Table 2).

**Table 2:** Logistic regression results of potential factors associated with the presence of acrochordon

***p*** value		%95 Confidence interval	
		Upper limit	Lower limit
**BMI**	0.743	0.827	1.306
**IGF-1R**	0.337	0.175	162.59
**IGF-2R**	0.996	0	
**Insulin**	0.179	0.554	1.11

The evaluation of variables (HOMA-IR, BMI, insulin and IGF-1) in terms of their effects on the presence/absence of IGF-1R and IGF-2R staining showed that IGF-1, insulin and HOMA-IR values were independently associated with staining (Table 3).

**Table 3:** The logistic regression analysis of independent variables associated with IGF-1R and IGF-2R staining

***p*** value		%95 Confidence interval	
		Upper limit	Lower limit
**IGF-1R staining**			
**HOMA-IR**	0.021	0.027	0.749
**BMI**	0.314	0.945	1.191
**Insulin**	0.020	1.100	2.987
**IGF-1**	0.032	0.976	0.999
**IGF-2R staining**			
**HOMA-IR**	0.023	0.023	0.758
**BMI**	0.084	0.985	1.275
**Insulin**	0.022	1.091	3.114
**IGF-1**	0.039	0.977	0.999

## Discussion

In the present study, metabolic parameters, serum IGF–1 level and tissue staining of IGF–1R and IGF–2R were investigated to evaluate their relationships with acrochordon. Mathur and Bhargava have reported that acrochordon and acanthosis nigricans may develop in patients with obesity-related insulin resistance and have shown that BMI and acrochordons count are correlated [9]. In a study conducted by Demir et al. to investigate the relationship between acrochordons and impaired carbohydrate metabolism, the mean BMI values of patients was 33.2 ± 6 kg / m2, and obesity was determined in 70% of their study population. They also reported that the number of acrochordons increased as BMI value increased (r = 0.36, *p* < 0.01) [2]. In our study, the mean BMI value of the acrochordon group was significantly higher than the control group; however, there was no correlation between BMI and acrochordon count. In another study that reported a similar relationship with BMI values, it was suggested that the higher frequency of acrochordons in the neck, axilla, and extremities could be associated with increased friction due to changes in body shape [10]. The lack of correlation between BMI values and acrochordon count in our findings may be due to various reasons, including age variation from other studies, the fact that all patients with at least one acrochordon were included in the study (which may skew results to the lower end of the spectrum), and also the limited number of patients and the relatively large age variance.

Insulin resistance, hyperinsulinemia, IGF-R activation, and obesity are closely related with each other. In this context, Bhargava et al. suggested that obesity, multiple acrochordons, abnormal glucose tolerance, pseudoacanthosis nigricans, and seborrheic keratoses may be components of a syndrome [11]. In our previous study, we had included 113 patients with acrochordon and had determined insulin resistance in 21.2% of these patients [12]. In a study by Sudy et al., the frequency of DM, impaired glucose tolerance and hyperinsulinemia/insulin resistance among males with 8 or more acrochordons was 11.5, 34.6 and 30.7%, respectively. Therefore, they suggested that having a high number of acrochordons (> 8) may be a cutaneous manifestation of hyperinsulinemia and pre-diabetic status [13]. In a study conducted by Tamega et al., it was suggested that acrochordons were associated with fasting insulin levels rather than fasting glucose levels [7]. Similarly, we found that fasting insulin level was significantly higher in the group with acrochordon compared to the control group. However, there was no statistically significant correlation between the number of acrochordons and insulin level.

The signal cascade following the activation of the IGF-2R receptor is not fully known. It is thought that the task of the IGF-2R receptor is to decrease the serum level of IGF-2 and its binding with IGF-1R. Thus, IGF–2R is thought to suppress tumor growth and proliferation by indirectly suppressing IGF-1R activation [8]. The first study that examined the relationships between acrochordon development and IGF-1 and insulin parameters (in non-diabetic individuals) was conducted by Jowkar and colleages. In this study, serum insulin was found to be higher in patients with acrochordon, whereas IGF-1 levels were similar [1]. In our study, serum IGF–1 values were also found to be comparable between thetwo groups. It has been suggested that acrochordons may result from dermal fibroblast proliferation by activation of IGF-1R [12]; however, there is no previous study to clarify this issue. Our study is the first to investigate IGF-1R and IGF-2R levels in acrochordon tissues. We believe that the role of IGF-R activation is more important than the increase in serum IGF-1 level and hyperinsulinemia in the acrochordon development process. In our study, IGF-1R and IGF-2R levels were significantly higher in the acrochordon group compared to the control group (*p* < 0.001). In patients with acrochordon, the intensity of IGF-1R staining was positively correlated with serum IGF-1 level and BMI. These results indicate that an increase in BMI may directly be associated with the presence of IGF receptor and intensity of receptor staining in tissue. The higher levels of BMI among patients in our study was higher, which supports this suggestion. We also found that there was a significant correlation between serum insulin levels and IGF-2R staining intensity. High insulin levels may directly affect the presence and intensity of IGF receptors, leading to the development of acrochordons. The presence of both IGF–1R and IGF–2R staining were found to be significantly more frequent in normoglycemic patients with acrochordon compared to controls. These results suggest that the levels of IGF receptors may play a role in the pathogenesis of acrochordon, regardless of impairment in glucose metabolism.

In our study, we found that IGF-1 level was the most important variable independently associated with the presence of staining with IGF–1R and IGF–2R. The effects of IGF–1 on IGF–1R are known. However, we think that the increase in IGF–2R levels may be due to negative feedback secondary to the increase of IGF–1. We also found that increased levels of HOMA-IR, serum IGF–1 and serum insulin are potential independent variables associated with the presence of both IGF–1R and IGF–2R. The high IGF receptor concentration in normoglycemic subjects and the independent association of HOMA-IR with the presence of receptor staining suggest that insulin resistance is independently associated with the presence and intensity of IGF receptors.

The results of our study have suggested that IGF–1R and IGF–2R may play role in the pathogenesis of acrochordon. We found that IGF receptor levels are correlated with BMI, serum insulin and IGF–1 levels in patients with acrochordon. When taken together, our results suggest that hyperinsulinemia and insulin resistance play a role in the development of acrochordon at tissue receptor level, and could be mediated by IGF–1. We believe that the etiopathogenesis of acrochordon can be elucidated if future studies confirm our findings and elucidate further factors affecting the presence and intensity of tissue IGF receptors.

## Data Availability

The data set forth in this publication is not publicly available and can be obtained from the corresponding author on request. The administrative permission to Access the data was obtained from HGK.

## Abbreviations

IGF: Insulin-like growth factor
IGF–1R: Insulin-like growth factor receptor–1
IGF–2R: Insulin-like growth factor–2
IGFBP: Insulin-like growth factor binding protein
BMI: Body mass index
HOMA-IR: Homeostatic Model Assessment-Insulin Resistance
DM: Diabetes mellitus
WHO: World Health Organisation
TINIA: Turbidimetric immun inhibition assay
ECLIA: Electrochemiluminescence immunoassay
FFA: Non-esterified fatty acid
NEFA-HR: Enzymatic colorimetric method
OGTT: Oral glucose tolerance test
IGT: Impaired glucose tolerance
HDL: High density lipoprotein
LDL: Low density lipoprotein
VLDL: Very low density lipoprotein
